# Modeling global 80-80-80 blood pressure targets and cardiovascular outcomes

**DOI:** 10.1038/s41591-022-01890-4

**Published:** 2022-07-18

**Authors:** Sarah J. Pickersgill, William T. Msemburi, Laura Cobb, Nicole Ide, Andrew E. Moran, Yanfang Su, Xinpeng Xu, David A. Watkins

**Affiliations:** 1 Department of Global Health, University of Washington, Seattle, WA USA; 2 Division of Data, Analytics, and Delivery for Impact, World Health Organization, Geneva, Switzerland; 3 Resolve to Save Lives, New York, NY USA; 4 Columbia University Irving Medical Center, New York, NY USA; 5 School of Public Health, Nanjing Medical University, Nanjing, China; 6 Department of Medicine, Division of General Internal Medicine, University of Washington, Seattle, WA USA

**Keywords:** Health policy, Hypertension

## Abstract

As the leading cause of death worldwide, cardiovascular diseases (CVDs) present major challenges for health systems. In this study, we analyzed the effects of better population blood pressure control in the context of a proposed 80-80-80 target: 80% of individuals with hypertension are screened and aware of their diagnosis; 80% of those who are aware are prescribed treatment; and 80% of those on treatment have achieved guideline-specified blood pressure targets. We developed a population CVD model using country-level evidence on CVD rates, blood pressure levels and hypertension intervention coverage. Under realistic implementation conditions, most countries could achieve 80-80-80 targets by 2040, reducing all-cause mortality by 4–7% (76–130 million deaths averted over 2022–2050) and slowing the rise in CVD expected from population growth and aging (110–200 million cases averted). Although populous middle-income countries would account for most of the reduced CVD cases and deaths, low-income countries would experience the largest reductions in disease rates.

## Abstract (web summary)

A new modeling study demonstrates that most countries could achieve 80-80-80 hypertension targets by 2040, promoting increased global equality for cardiovascular diseases with the greatest mortality benefits being seen in low-income countries.

## Main

Since the 1990s, healthcare advances have generated considerable declines in mortality and birth rates, accelerating population growth and aging^1,2^. In parallel, risk factors for non-communicable diseases (NCDs)—including CVD, which is now responsible for a third of all deaths worldwide—are becoming much more prevalent, in part due to economic development and globalization^3–5^. The number of CVD cases and deaths has continued to rise because population growth and aging have outpaced declines in age-specific CVD incidence and mortality from public health and clinical interventions^2^. These unfavorable composite trends are expected to continue, particularly in low-income countries (LICs) and middle-income countries (MICs) where demographic drivers of CVD are the strongest^6,7^. Furthermore, health systems in many of these countries face considerable resource constraints, especially in providing advanced CVD care.

Raised blood pressure (hypertension) is the leading modifiable risk factor for death and disability worldwide, and several blood pressure reduction interventions are very cost-effective in LIC and MIC settings^8–10^. Improved population blood pressure control has the additional benefit of reducing demand for CVD care, which is particularly important in countries where referral and specialized care systems are less developed^11^. Unfortunately, hypertension is not high on national policy agendas for several reasons. For example, the health and economic benefits of CVD prevention can take many years to accrue, with no obvious short-term incentives to drive policy change. In addition, many in the global health and development assistance communities continue to perceive NCDs as diseases of affluence, and investment in areas such as CVD is perceived as anti-equity^12^. Similar challenges and misconceptions have plagued the broader NCD agenda, which has also suffered from chronic political neglect^13^.

In this study, we model the health benefits of better population blood pressure control in 182 countries (Extended Data Table 1) and conduct several additional analyses to put these benefits in the broader global health context. Our overarching objective is to establish reasonable boundaries on what could be achieved if hypertension were to become a political priority and if there were international collective action to support scale-up of interventions in different countries. The scenarios we model are informed by successful hypertension programs and best-performing countries, which, if replicated around the world, could accelerate reductions in CVD compared to maintaining the status quo.

Within this overarching objective, we look at the likelihood of achieving an 80-80-80 target for global hypertension: 80% of individuals with hypertension are screened and aware of their diagnosis; 80% of those who are aware are prescribed treatment; and 80% of those on treatment have achieved guideline-specified blood pressure targets. This target is in the spirit of the 90-90-90 international target for HIV/AIDS^14^, another chronic health condition that can be readily tracked using the care cascade approach and that often implements similar solutions to improve outcomes in primary healthcare settings^15^. An 80-80-80 target for national hypertension programs might prove more practical than a 90-90-90 target for hypertension, because a much larger number of people are living with hypertension as compared to HIV, and some cases of raised blood pressure can be prevented or partly managed via dietary sodium reduction and other behavioral measures^16,17^. Most importantly, an 80-80-80 target implies that approximately 51% of individuals with hypertension would have controlled blood pressure, and this level of control has already been achieved nationally in some countries^18^ and in several subnational hypertension programs, including in LICs and MICs^19,20^.

Our modellng analysis is based on two approaches to population blood pressure control that are widely seen as providing good value for money and that have been successfully implemented outside of high-income country (HIC) settings^21^: (1) pharmacological treatment of hypertension to a goal systolic blood pressure (SBP) of either ≤140 mmHg or ≤130 mmHg in accordance with current guidelines for specific at-risk groups^16^, and (2) a World Health Organization (WHO)-endorsed package of population-wide policies to reduce the consumption of dietary sodium^22^. Using 2022 as the baseline year for our analysis, we estimate the effect of these two interventions on future CVD rates worldwide by 2050. We look at the four major types of CVD that are linked to raised blood pressure: ischemic heart disease (IHD), ischemic stroke, hemorrhagic stroke and hypertensive heart disease (HHD).

Throughout the paper, we consider three scenarios for intervention implementation:

1. **Business as usual**—hypertension control increases according to historically observed rates of change^18^, and population mean sodium intake values remain at their current levels. This scenario reflects the consequences of making no additional effort to increase the rate of scale-up for interventions between 2023 and 2050 and serves as a comparator for the latter two scenarios.
2. **Progress**—hypertension control accelerates to rates of improvement observed historically in high-performing countries (for example, about 3% per year at intermediate levels of intervention coverage); population mean sodium intake decreases by 15% between 2023 and 2030 (without falling below 2 g per day)^17,23^.
3. **Aspirational**—hypertension control accelerates to rates of improvement that are faster than observed historically in high-performing countries (for example, about 4% per year at intermediate levels of intervention coverage) but slower than the most successful global health program: anti-retroviral drug therapy for HIV (about 5% per year); and population mean sodium intake decreases by 30% between 2023 and 2027 (without falling below 2 g per day).

Justification for the assumed acceleration in the implementation of these two interventions in the progress and aspirational scenarios is provided in the Methods. The values we choose reflect contemporary experiences with sodium reduction and hypertension treatment scale-up in a range of countries.

## Results

The principal findings of our analysis, aggregated by World Bank country income group, are presented here. Additional results for specific countries, and disaggregated results by age, sex and specific type of CVD, are available for download through an online data visualization tool (https://dcp-uw.shinyapps.io/bp-targets/).

### Reduced blood pressure would substantially improve population cardiovascular health

**Fig. 1:**
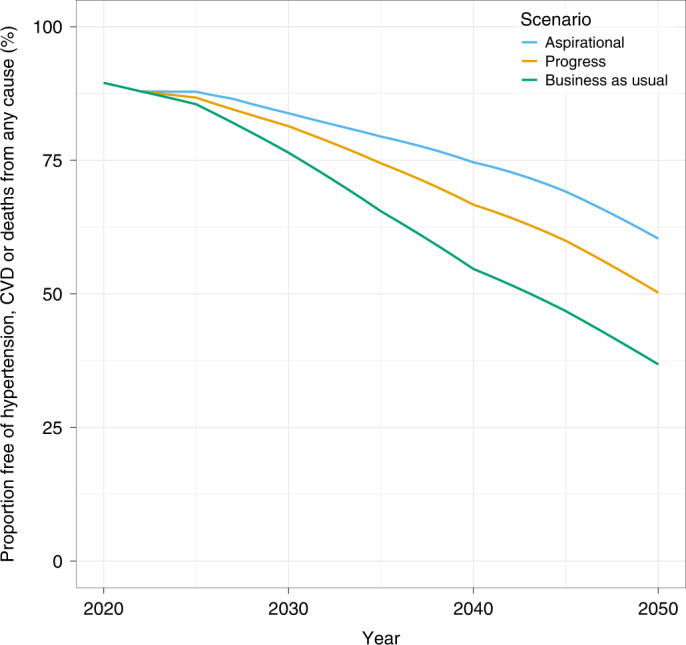
Evolution of cardiovascular health status in a cohort of at-risk adults, across three blood pressure intervention scenarios. The probability of remaining free of hypertension, CVD or all-cause death among an example cohort of individuals aged 35–39 years in 2020 (global population, both sexes) as they age from 35–39 years in 2020 to 65–69 years in 2050.

Figure 1 illustrates the improvement in cardiovascular health—defined as the probability of remaining free of hypertension, CVD or death from any cause—for the cohort of adults aged 35–39 in the year 2020 and who would be aged 65–69 in 2050. In this cohort, the proportion of adults without raised blood pressure or CVD would decline over time, reflecting increased risks associated with age. However, this proportion would decline more slowly, with 50% (progress scenario) and 60% (aspirational scenario) of individuals alive and without hypertension or CVD in 2050 as compared to 37% in the business-as-usual scenario. Reciprocally, the proportion of the cohort affected by hypertension or CVD would rise more slowly in the progress and aspirational scenarios, relative to the business-as-usual scenario.

### Improved cardiovascular health would markedly decrease CVD incidence and death rates

Table 1 summarizes the worldwide consequences of achieving better global population blood pressure control in the coming decades. In the business-as-usual scenario, the absolute number of CVD deaths would increase from 17 million in 2020 to 37 million in 2050, and the absolute number of CVD cases would increase from 320 million to 710 million. Compared to this scenario, 2.8 million CVD deaths and 4.1 million new cases per year on average, or 76 million deaths and 110 million new cases in total, could be averted by 2050 in the progress scenario. In the aspirational scenario, 4.8 million CVD deaths and 7.4 million new cases per year on average, or 130 million deaths and 200 million new cases in total, could be averted by 2050. Relative to the business-as-usual scenario, these two scenarios represent a 13% and 22% relative reduction in CVD deaths, respectively, and a 7.1% and 13% relative reduction in new CVD cases.

**Table 1:** Projected worldwide health consequences of blood pressure intervention scenarios

	Business as usual (2020)	Business as usual (2050)	Progress (2050)			Aspirational (2050)		
			Sodium reduction	Hypertension treatment	Both interventions	Sodium reduction	Hypertension treatment	Both interventions
Number of CVD deaths (millions)	17	37	36	33	33	36	30	29
Difference	*-*	*-*	−1.9%	−11%	−13%	−3.7%	−20%	−22%
Number of all-cause deaths (millions)	50	82	81	79	79	81	76	76
Difference	-	-	−0.57%	−3.4%	−3.8%	−1.1%	−6.3%	−6.8%
Number of new CVD cases (millions)	35	76	74	71	70	73	67	66
Difference	-	-	−1.7%	−5.8%	−7.1%	−3.3%	−11%	−13%
Number of prevalent CVD cases (millions)	320	710	700	690	680	690	670	660
Difference	-	-	−1.9%	−2.8%	−4.3%	−3.7%	−5.5%	−7.8%
Life expectancy at birth (years)	73	78	78	78	78	78	79	79
Difference	-	-	0.11%	0.70%	0.78%	0.22%	1.2%	1.3%
Probability of dying between 30 years and 80 years of age from CVD	0.20	0.18	0.18	0.15	0.15	0.17	0.14	0.13
Difference	-	-	−2.3%	−14%	−16%	−4.5%	−24%	−26%

Aggregate estimates of the effect of improved blood pressure control in 182 countries. Results are based on estimates of CVD deaths and cases across all age groups (20+ years) and both sexes. For the purpose of reporting the results in this table, the effects of sodium reduction and hypertension treatment were modeled separately and then together (‘both interventions’). The difference reported is the relative difference of the intevention scenario compared to the business-as-usual projections for 2050.

About 4–7% of projected all-cause deaths in the year 2050 could be prevented in the progress and aspirational scenarios, respectively, as compared to the business-as-usual scenario. Given that most of the CVD deaths that would be averted through improved hypertension control would occur at older ages, life expectancy at birth would increase by a modest 0.78–1.3%; however, remaining life expectancy at age 40 would increase by 1.5–2.6%, or 0.63–1.1 years.

As described in the Methods, we also looked at changes in a summary measure of cardiovascular health: the probability of dying from any CVD cause between the exact ages of 30 and 80 (that is, CVD-specific 50q30). This probability would be reduced by 16% and 26% in the progress and aspirational scenarios, respectively.

As suggested in Table 1, about 85–83% of the potential reduction in mortality would result from scale-up of hypertension treatment in the progress and aspirational scenarios, respectively, with the remaining 15–17% from sodium reduction. Among the four specific causes of death we modeled, IHD would comprise the largest proportion of cases and deaths averted globally, although this would vary by region, with hemorrhagic stroke being relatively more important in sub-Saharan Africa and East Asia than in other regions. Across scenarios and regions, health gains would be similar for men and women. Most of the averted CVD cases and deaths would be in MICs in South and East Asia where most of the world population lives. The online data visualization tool provides additional findings disaggregated by age group, sex, cause of death and location, as well as disaggregated estimates for sodium reduction alone and for hypertension treatment alone.

### Blood pressure interventions could partly counteract the demographic drivers of CVD

**Fig. 2:**
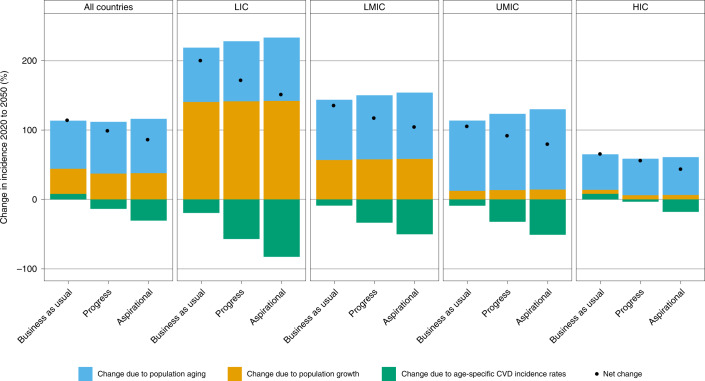
Demographic decomposition of the change in new CVD cases in three blood pressure intervention scenarios between 2020 and 2050, by country income. The three factors that determine the change in the number of new CVD cases (*y* axis) are population aging, population growth and changes in age-specific CVD incidence rates. The former two are often referred to as demographic drivers; the latter is often referred to as epidemiological change. The net effect of these three factors on changes in CVD incidence is represented by the black points. LMIC, lower-middle-income countries; UMIC, upper-middle-income countries.

Because of population growth and aging, the number of non-fatal and fatal CVD cases will approximately double between 2020 and 2050 (Table 1). Figure 2 presents a decomposition analysis of the projected growth in the number of new CVD cases between 2020 and 2050 across the three scenarios. Findings are disaggregated by country income group and underscore the differences in underlying demographic drivers across different settings. For example, LICs currently have the highest all-cause mortality worldwide but are projected to continue making progress on mortality reduction, so population growth will be the principal driver of CVD incidence. By contrast, MICs (especially upper-MICs) and HICs have lower all-cause mortality and are projected to make more progress on birth rate reduction, so population aging will be the principal driver of CVD incidence^24^.

**Extended Data Fig. 1:**
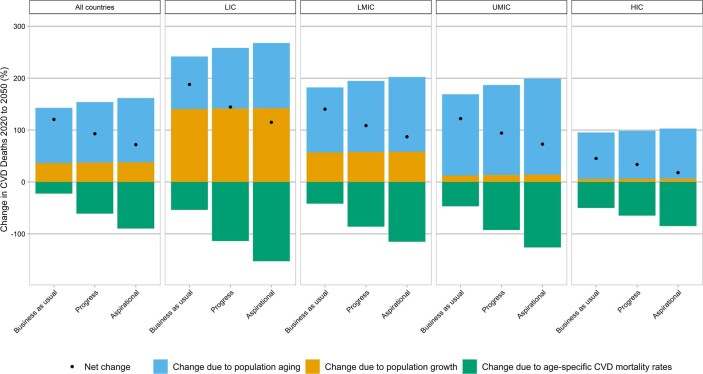
Demographic decomposition of the change in new CVD deaths, by country income, over 2020–2050. The results presented here are analogous to those presented in the main text but with mortality, rather than incidence, on the y axis.

Reducing mean population blood pressure would reduce the incidence of CVD at any given age, partially counteracting the effects of population growth and aging (Fig. 2). In relative terms, the effect of blood pressure reduction on the growth rate in the number of CVD deaths would be similar to its effect on CVD incidence (Extended Data Fig. 1). Although the total population of LICs is relatively small compared to MICs (0.67 billion versus 5.8 billion in 2019) and, hence, the total number of CVD cases and deaths is relatively small, LICs would experience a larger reduction in the growth rate in CVD. Given that HIC populations generally have higher levels of blood pressure control and already have lower age-specific CVD rates, the reductions in the growth rate in CVD would be the smallest in these countries.

### Scale-up of blood pressure interventions could reduce CVD inequalities across countries

**Fig. 3:**
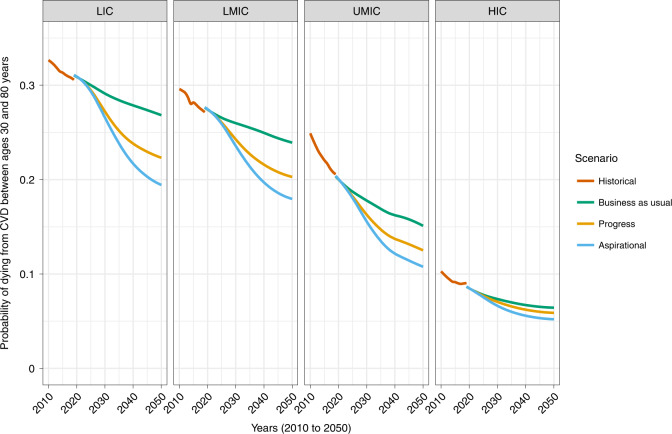
Long-term trends in CVD-specific 50q30 from 2010 to 2050 in three blood pressure intervention scenarios, by country income. Projections of population size and structure beyond 2019 factor in the demographic effects of excess mortality from the COVID-19 pandemic, but this does not considerably change mortality probabilities or long-term age-specific CVD death rates. LMIC, lower-middle-income countries; UMIC, upper-middle-income countries.

At present, LICs and lower-MICs have the highest CVD mortality as measured by CVD-specific 50q30 (Methods). If historical trends continue, these inequalities will persist, because rates of decline in CVD mortality have generally been slowest in the poorest countries^25^. Implementation of the two blood pressure interventions, and achievement of an 80-80-80 target, would have the greatest benefit in these countries (Fig. 3). For example, LICs would experience a 0.29–0.38% average annual decline between 2022 and 2050 in the progress and aspirational scenarios, respectively, as compared to 0.13% in the business-as-usual scenario. By contrast, the decline in HICs would be about 0.09–0.11% in the progress and aspirational scenarios, respectively, as compared to 0.07% in the business-as-usual scenario.

In absolute terms, cross-country differences in CVD mortality probability would lessen appreciably by 2050. In 2022, CVD-specific 50q30 was 0.31 in LICs and 0.085 in HICs, an absolute difference of 0.22. By 2050, in the business-as-usual scenario, this difference would reduce to 0.20, whereas this gap would reduce to approximately 0.16 in the progress scenario and 0.14 in the aspirational scenario. Put another way, 20–30% of the difference in CVD mortality between LICs and HICs could be eliminated through better blood pressure control alone.

### An 80-80-80 target could be achieved in all countries between 2040 and 2050

**Fig. 4:**
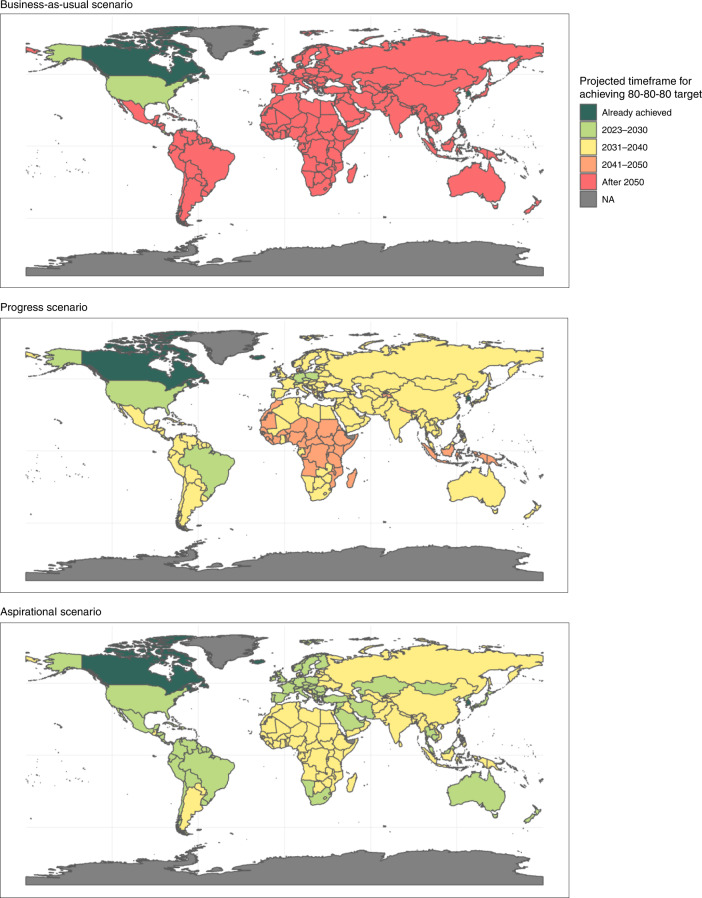
Year by which an 80-80-80 target would be achieved in each country. The three panels present results for the three blood pressure intervention scenarios outlined in the article.

**Extended Data Fig. 2:**
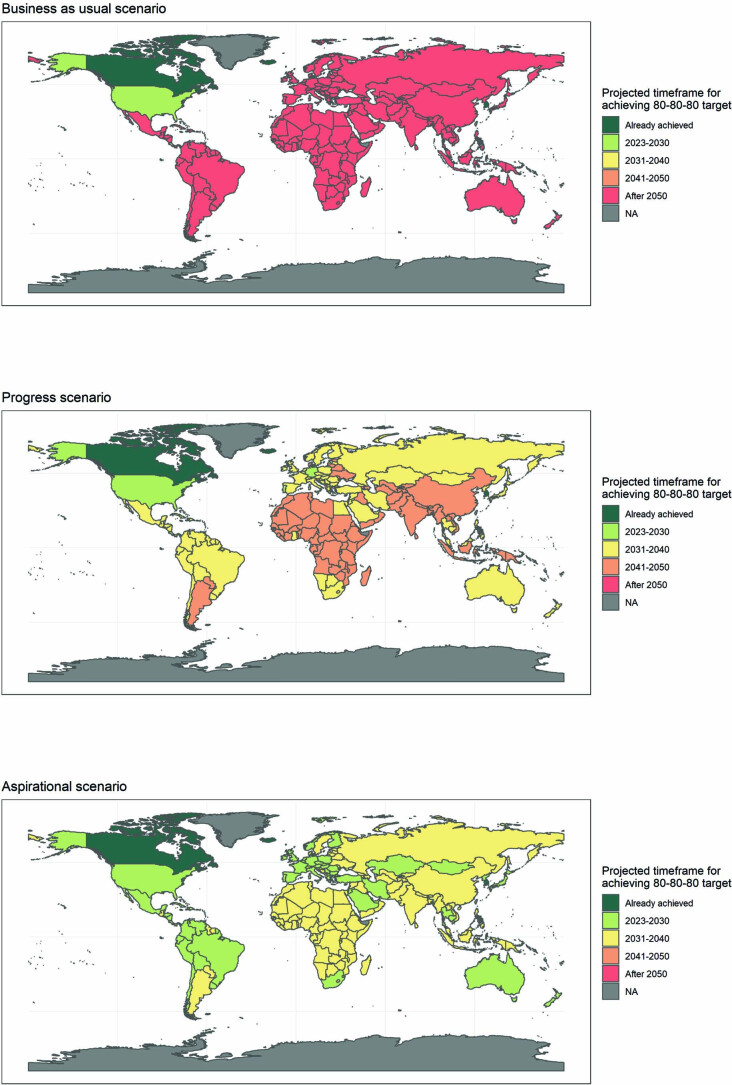
Consequences of no additional action on sodium intake. The three panels illustrate the projected year by which the 80-80-80 target for hypertension control would be achieved without co-implementation of sodium reduction policies.

Figure 4 presents country-specific estimates of the year by which the blood pressure target would be achieved in each of the three scenarios. Most countries would not achieve the 80-80-80 target until after 2050 in the business-as-usual scenario, and some countries would only achieve the target after 2070. By contrast, the progress scenario would involve an acceleration of current trends that would be sufficient for all countries to achieve the target by 2050, with many countries achieving the target by 2040, except for some in Africa and Asia. In the aspirational scenario, all countries would achieve the target by 2040. Implementing sodium reduction policies would be important to achieving the target. Without these policies—and relying solely on individual clinical management of high blood pressure—countries would require an additional 1–3 years on average in the aspirational and progress scenarios, respectively (Extended Data Fig. 2).

## Discussion

The Coronavirus Disease 2019 (COVID-19) pandemic has disrupted health systems around the world, and NCD control programs, which usually include hypertension screening and care, have been among the most affected^26^. We demonstrate that an 80-80-80 target for blood pressure control, although ambitious, is achievable in most countries by 2040 and could be a key component of the post-COVID-19 NCD agenda. Tens of millions of lives could be saved in the process, and global inequalities in CVD would be substantially reduced. Rapid scale-up of CVD prevention interventions has proven elusive in LICs and MICs, although there have been notable successes in the past decade^19,20^, and the weaknesses in public health and primary health care revealed by the COVID-19 pandemic could spur health system reforms and attempts to replicate these successes elsewhere.

In most countries, the largest relative gap in the cascade of hypertension care is between the proportion on treatment and the proportion whose blood pressure is at goal^18^. This metric roughly corresponds to the ‘quality’ of hypertension care locally. Intensive screening programs can reduce population blood pressure and help to achieve an 80-80-80 target, but they depend on high-quality healthcare further down the cascade^27^. Improving quality will involve measures that address supply-side gaps, like provider-level and organizational-level fidelity to simple guidelines—such as those developed for the HEARTS program^19,28^—and access to affordable medicines and healthful foods, complemented by measures that address demand-side gaps, like patient retention in care and medication adherence. Other innovations in hypertension service delivery have been developed and could further enhance quality, but they require further study and proof of effectiveness at scale. Examples include digital solutions like mobile tracking applications built on the widely used DHIS2 platform^29,30^ and drug reformulations like novel fixed-dose combinations of anti-hypertensive drugs^31^.

Our analysis suggests that reducing population blood pressure, through a combination of dietary sodium reduction and better hypertension treatment, is one of the single most impactful measures that can be taken to improve global public health. We estimated that scaling up these two complementary public health and clinical interventions could prevent between 76 million and 110 million deaths from CVD and between 130 million and 200 million new cases of CVD between 2022 and 2050. Putting these numbers in context, the effect on all-cause mortality (−3.8% to −6.8%) would be proportionally the same as if the world were to completely eliminate HIV/AIDS, tuberculosis, malaria and maternal mortality (about 5% of deaths combined in 2019)^2,32^. Importantly, implementing these blood-pressure-lowering interventions would considerably improve population cardiovascular health, even after accounting for inevitable increases in CVD due to population growth and aging.

Our analysis also provides counterevidence to the presumption that CVD prevention interventions only serve the needs of the wealthiest. In fact, we demonstrate that the poorest countries stand to gain the greatest benefit from progress on hypertension control, because these are generally the countries with the highest CVD mortality rates and lowest coverage of blood pressure interventions. Fully implementing these two interventions could more than double the rate of decline in CVD mortality in the poorest countries. Population growth and aging will continue to drive increases in the number of individuals with fatal and non-fatal CVD, but improved population blood pressure control could substantially reduce demand for specialized cardiovascular care, saving precious resources and, potentially, strengthening the capabilities of primary healthcare systems to deliver high-quality care for a broader range of chronic illnesses^25^.

Despite the magnitude of the problem, very few studies have attempted to look at the potential effect of blood pressure interventions at a cross-country or global level^33–35^. In addition, little is known about the population effects of CVD interventions on outcomes other than mortality or on the distribution of these outcomes across populations. Here we present a range of cardiovascular health metrics that might be considered as part of an expanded post-Sustainable Development Goal NCD agenda^36^. Such a CVD dashboard (https://dcp-uw.shinyapps.io/bp-targets/) might include trends in the proportion of the population in ideal cardiovascular health (Fig. 1), incidence of non-fatal CVD, mortality probability across broad age groups (including consideration of individuals over 70 years of age) and—importantly, for collective action—inequalities in CVD outcomes across countries. Efforts to develop country CVD dashboards would need to be integrated alongside dashboards for infectious and maternal/child diseases and supported by investments in surveillance and vital registration systems, particularly in sub-Saharan African countries^37^.

Our modeling approach offers several innovations and substantial advantages over previous reports on global hypertension and CVD. The blend of demographic and state-transition modeling and calibration of our model to Global Burden of Disease Study^2^ and United Nations Population Division^38^ estimates allowed us to generate plausible future projections for countries and regions that have external comparability and validity. We specifically emphasize changes in non-fatal CVD, which are of particular interest to policymakers tasked with preparing budgets and allocating resources across a range of health programs. Our analyses seek to provide a fresh perspective on the urgency of CVD prevention in LICs and MICs and to understand the effect of these interventions on global cardiovascular health inequalities. Additionally, and in contrast to counterfactual approaches (for example, comparative risk assessment methodology), our modeling strategy is consistent with cohort-based evidence and allows for projection of age-specific and sex-specific outcomes that can be etiologically attributed to raised blood pressure^39^.

Despite its strengths, our study also has limitations. We focused on two interventions that are strongly linked to CVD outcomes. Other behavioral risk factors, such as non-sodium dietary components, smoking and alcohol use, can affect blood pressure control at the margin, but we did not incorporate interventions targeting these risk factors in our analysis. Notably, raised blood pressure is only one risk factor for CVD. Making greater progress on CVD mortality would require analysis of other interventions, such as tobacco and high cholesterol control, diabetes and obesity interventions and better secondary prevention for individuals living with chronic CVD. These analyses were outside the scope of our study but would provide a fuller picture of the opportunities for reduced CVD incidence and mortality. We also note that sodium reduction has benefits to outcomes other than CVD, such as gastric cancer, although these effects are small at a population level^40^.

We conclude that achieving an 80-80-80 target for hypertension control could be one of the single most important global public health accomplishments of the coming decades. Achieving this target on a fast track (by about 2040) will require replication of successful hypertension programs at scale in many countries, complemented by rollout of WHO-supported sodium reduction policies. These efforts will, in turn, require international collective action to develop new technologies, share best practices and, in some cases, counter industry resistance to food reformulation policies. Implementing these two hypertension interventions is an urgent priority for national health systems and for the global health community. Successful implementation would partially counter the expected increase in CVD due to population growth and aging, reducing demand for costly CVD treatments, and would also reduce global inequalities in CVD outcomes between poorer and wealthier countries.

## Methods

Our modeling approach blended state-transition modeling methods with demographic projection methods described in detail below. This hybrid approach allowed us to model the evolution of population size, population structure, fatal and non-fatal CVD events and death from non-CVD causes worldwide over the period 2020–2050 for adults aged 20–95 years in 182 United Nations Member States, categorized by World Bank income level (Extended Data Table 1). Outcomes were modeled for four specific CVD causes: IHD, HHD, ischemic stroke and hemorrhagic stroke. In this paper, we use the term ‘CVD’ to refer to the combined results for IHD, HHD, ischemic stroke and hemorrhagic stroke. These four causes were identified as having the strongest evidence of association with reduced blood pressure and comprise most of the health loss from CVD^2,41^. We include HHD in this analysis because, although IHD and stroke account for over 90% of deaths from CVD, HHD is particularly important in regions like sub-Saharan Africa where the prevalence of hypertension is high but the prevalence of other risk factors like smoking and obesity is comparatively lower.

This analysis did not require ethics approval because no human subjects were part of the research.

### Model structure

We used four state-transition (Markov) models, one for each specific CVD cause, to capture the dynamic relationship among disease incidence, prevalence and mortality as well as competing risk from other causes of death. Each cause model contained four mutually exclusive states: ‘well’, ‘sick’, ‘dead’ and ‘dead (other)’. Disease-specific prevalence and deaths over time were captured in the four independent models. For each 1-year time step, *t*, and cause, *c*, the number of prevalent cases (or those in the ‘sick’ state) for each age, sex and country was defined as

1 $$sick_{t,c} = sick_{t - 1,c} \ast \left( {1 - \left( {CF_{c} + BG_{mx,t}} \right)} \right) + well_{t - 1,\,c} \ast IR_{c}$$

where CF_c_ is the cause-specific transition probability from sick to dead from cause *c* (case fatality); BG_mx,t_ is the cause-specific transition from sick to dead from any non*-c* cause (background mortality); and IR_c_ is the transition from well to sick (incidence) from that cause (Supplementary Fig. 1).

Similarly, the number of cause-specific deaths for each age, sex and country was defined as

2 $$dead_{t,c} = sick_{t - 1,c} \ast CF_{c}$$

Other-cause mortality risk was factored in at the aggregate level at the end of each time step to ensure the integrity of the all-cause death envelope (see ‘Statistical analyses’ below)

3 $$pop_t = pop_{t - 1} - all\,cause\,deaths_{t - 1}$$

4 $$all\,cause\,deaths_t = IHD\,deaths_t + HHD\,deaths_t + stroke\,deaths_t + pop_t \ast BG_{mx,t}$$

where BG_mx,t_ is the mortality rate for all causes except IHD, HHD, ischemic stroke and hemorrhagic stroke. Then, the population in the ‘well’ state was adjusted accordingly:

5 $$well_{t,c} = pop_t - all\,cause\,deaths_t - sick_{t,c}$$

The four state-transition models were integrated within a demographic model that used a cohort component projection approach. Estimates of mortality rates by age, sex, specific CVD case and year were constrained to fit within the all-cause mortality envelopes implied by the demographic projections. The business-as-usual scenario in this model reflects a projection forward of secular trends in rates of change in all-cause mortality, fertility and migration, calibrated to World Population Prospects 2019 (ref. ^38^) (WPP 2019) data by age, sex and country. Intervention effects in the progress and aspirational scenarios (see below) are reflected in lower CVD-specific incidence and mortality rates that reduce all-cause mortality relative to the business-as-usual scenario and have knock-on effects on population size and structure over time.

Formally, the cohort component projection is expressed as follows:

6 $$P_{t + 1} = L\left( {P_t + 0.5 \times I_t} \right) + 0.5 \times I_t$$

where $P_i$ is the vector of age-specific and sex-specific population numbers at time *i*; $L_i$ is the Leslie matrix comprising of estimates of age-specific fertility in the first row and survival proportions in the off-diagonal; and $I_t$ gives the number of net migrants estimated relative to the population.

### Statistical analyses

We first estimated transition probabilities in each of the four CVD cause models by calculating the average annual rate of change in disease-specific prevalence and mortality by 5-year age group, sex, cause and location using data from the Global Burden of Disease Study^2^ (GBD) 2019. These rates of change were applied to the state-transition models over the ‘observed’ period 2014–2019 to calculate annual transition probabilities by 1-year age group. We compared our modeled results for 2010–2019 with GBD estimates of CVD prevalence and mortality by country and sex and then applied post hoc adjustment factors to the transition probabilities to calibrate our model to GBD 2019 estimates. The calibration process used a composite measure of root-mean-squared errors for deaths and prevalence that were minimized for each country–cause pair; the composite measure placed greater weight on the error in mortality estimates, which are generally more reliable than prevalence estimates. The resulting adjusted transition probabilities were used in our projection model for the start year 2019.

For 2020–2050, transition probabilities to the other-cause death state (that is, background mortality) and from the diseased state to the CVD-specific death state (that is, CVD case-fatality) were adjusted to reflect the continuation of historical rates of change in case-fatality and other-cause mortality from the 2010–2019 period (implied by the GBD 2019 trends) to reflect secular advancements in care. The transition probabilities from the well state to the diseased state were assumed to be constant over time, which was consistent with GBD 2019 age-specific CVD incidence trends, whereby any reductions in incidence would be captured by in the increase in blood pressure control rates described in the intervention scenarios.

We then parameterized and calibrated the demographic projection model with data from WPP 2019, which provide age-specific fertility and sex–age-specific mortality rates by 5-year age groups and for 5-year periods in their projections. WPP 2019 provides annual interpolated estimates of (1) single-age-specific population by sex, (2) total births and (3) total deaths by sex. Calibration of our model to WPP 2019 was as follows:

- Splines were used to smooth the quinquennial mortality and fertility rates to single ages and single years.
- Sex–age-specific counts for deaths and sex-specific births were calculated for specific years based on applying the rates to the population.
- Rates were rescaled based on the ratios of the aggregates generated from the population to the interpolated aggregates from WPP 2019.
- Migration rates by age and sex were derived as the residual component when balancing the population change between consecutive years with the change implied by the estimated births and deaths by sex and age.

For simplicity and transparency, we assumed zero net migration for every country included in the analysis. We also did not factor in changes in fertility rates because these would not affect the population at risk of CVD between 2020 and 2050.

As of the date of this analysis, global health and demographic estimates were available only through the year 2019. Our projection model generated estimates for 2020–2022, the years just before intervention implementation was assumed to begin. Because it was based on WPP 2019 and GBD 2019 input data, it did not account for the effect of COVID-19 on current and future population size and structure. We did a post hoc adjustment of our projections based on observed excess mortality during the pandemic. Specifically, we took data from Eurostat and WHO (https://covid19.who.int/WHO-COVID-19-global-data.csv) on the number of excess all-cause deaths by country and subtracted these from the counts of populations remaining in the model in the years 2021–2022 (see Equations 4–6). In the absence of data to suggest otherwise, excess deaths were assumed to follow the same age pattern as all-cause deaths in WPP 2019.

To further validate our calibration procedures, we compared our model projections for the business-as-usual scenario to the WPP 2019 age-specific population projections through 2050 and GBD 2016 Study projections of trends in CVD-specific, age-standardized mortality rates through 2040. These are included in the online data visualization tool: https://dcp-uw.shinyapps.io/bp-targets/.

To estimate the effect of reduced population blood pressure on CVD events, we took estimates of the relative risk of new CVD (specific to the degree of blood pressure elevation) from the literature^41^. These estimates were based on a meta-analysis of 123 trials that were undertaken in genetically and ethnically diverse populations, albeit predominately from HIC study sites. Observational data from LICs and MICs corroborate the strength of these associations^42^. Because relative risk data came from randomized controlled trials, they were assumed to reflect the marginal effect of blood pressure reduction, adjusted for other CVD risk factors like smoking and obesity. Relative risks were then weighted by the observed population blood pressure distribution (see Equations 7–9) and scaled to observed levels of CVD incidence to generate CVD incidence rates specific to the degree of blood pressure elevation. A leftward shift in the population blood pressure distribution would, therefore, reduce the proportion of the population at higher relative risk of CVD and, thus, reduce incidence rates in our intervention scenarios relative to baseline. This is illustrated in Supplementary Fig. 2.

Baseline blood pressure distributions were constructed assuming a normal distribution. Specifically, we used age-specific, sex-specific and country-specific mean SBP estimates from the NCD Risk Factor Collaboration (NCD-RisC)^43^ and age-specific and sex-specific 5th-percentile blood pressure levels in populations free of CVD and anti-hypertensive drug use^44^. The latter ranged from an average of 96 mmHg in females aged 20–39 years of age to an average of 118 mmHg in males over 80 years of age. This approach produced more stable variance estimates for the blood pressure distributions than an approach using mean systolic blood pressure and the proportion of the population with raised blood pressure as reported in NCD-RisC. However, our method produced estimates of the prevalence of raised blood pressure that showed excellent agreement with NCD-RisC estimates (intraclass correlation coefficient 0.941 (95% confidence interval: 0.929, 0.951)). Country-specific, age-specific and sex-specific distributions were then shifted according to the intervention scenario, and the resultant incidence rates were calculated based on a new distribution of blood pressure as described below.

For each blood pressure category (i), the incidence of CVD (y_i_) can be estimated using a ‘standardized’ CVD risk parameter (${\upalpha}$), which is calculated the taking the sum of blood-pressure-specific relative risks of CVD (RR_i_) times baseline (that is, no intervention) proportion of the population in each SBP category (p_i_). RR_i_ estimates for IHD, HHD and stroke were derived from the above-mentioned systematic review and meta-analysis on blood pressure lowering for prevention of CVD^41^. The average incidence irrespective of blood pressure (y) is then multiplied by the SBP-specific relative risk (RR_i_) divided by the risk parameter (${\upalpha}$) to give the blood-pressure-specific incidence rate of disease (y_i_).

7 $$\alpha = \mathop {\sum }\limits_{i = 1}^n p_i \times RR_i$$

8 $$y_i = \frac{{RR_i}}{\alpha } \times y$$

Then, the average incidence (y^*^) for the intervention scenario can be calculated by taking the population-weighted average using the intervention-adjusted proportion of population in each SBP category (p_i_^*^):

9 $$y^ \ast = \mathop {\sum }\limits_{i = 1}^n p_i^ \ast \times y_i$$

As mentioned previously, this approach allows for estimation of the marginal effects of blood pressure reduction in populations with multiple CVD risk factors. Consider the example of males aged 60–64 years in China. In our model, the incidence of IHD in this group in 2019 (post-calibration) was 556 per 100,000 person-years. If we were to instantaneously increase the proportion of this group whose blood pressure is controlled from 14% (estimate for 2019) to 100%, IHD incidence would decrease to 472 per 100,000 person-years, a 15% reduction. The remaining 85% of IHD incidence in this group is explained by other factors, such as smoking, high body mass index, dietary risks and raised blood pressure that is below the guidelines target of 140 mmHg but in excess of the theoretical minimum risk level of 110–115 mmHg^45^. Our model assumes that the residual incidence of 472 per 100,000 person-years in this age group would remain unaffected by the interventions and would be the risk experienced by subsequent cohorts as they age within our demographic model.

Effects of the sodium reduction intervention were calculated using estimates of a 1.12-mmHg reduction in SBP for every gram of reduced salt consumption for individuals with raised blood pressure and a 0.58-mmHg reduction in SBP for every gram of reduced salt consumption for individuals with normal blood pressure^46^. In each time step of the model, the blood pressure effect of sodium reduction is estimated before calculating the effect of increased coverage of hypertension treatment.

Current population dietary sodium intake was taken from the GBD 2019 Study^47^. The effects of sodium intake reduction on mean systolic blood pressure were taken from a systematic review^48^.

Better population blood pressure control not only affects the incidence of CVD; it also influences mortality among individuals with established CVD. We modeled the secondary prevention effects of scale-up of pharmacological treatment of blood pressure by applying estimates of relative risk reduction found from the literature to the case–fatality ratios (that is, transition from ‘sick’ to ‘dead’) in our state-transition models. Specifically, we assumed a 20% relative reduction in IHD mortality among the proportion of IHD cases (30%) affected by heart failure^49,50^ and a 26% reduction among those without heart failure^51^. We estimated a relative reduction in stroke mortality of 36% for ischemic stroke and 76% for hemorrhagic stroke^52^. Lastly, we assumed the same effect size for HHD as for IHD with heart failure (that is, 20%).

In addition to reporting counts and rates of CVD cases and deaths, we calculated summary population measures, including life expectancy and mortality probability, using standard demographic methods to generate aggregate life tables.

The Sustainable Development Goal indicator for NCD mortality (target 3.4) is the probability of dying of an NCD between the exact ages of 30 and 70 (that is, 40q30). The NCD indicator was recently extended to look at global progress on mortality from specific NCDs, including CVDs^53^. As compared to using death counts or crude death rates, using probability of death as an indicator allows for easy comparison of age-specific death rates across countries and over time. However, the upper age for the NCD target has been critiqued as too restrictive^54–56^. In this paper, we estimated the probability of dying between the ages of 30 and 80 (50q30), acknowledging that many individuals with CVD are in the 70–79-year age group and benefit from blood pressure reduction. To calculate 50q30, we first calculated age-specific mortality for each 5-year age group and then converted these rates into age-specific probabilities of death:

10 $${}_5q_x = \frac{{}_5M_x \ast 5}{{1 + {}_5M_x \ast 2.5}}$$

Mortality probabilities were then aggregated to estimate CVD-specific 50q30:

11 $${}_{50}q_{30} = 1 - \mathop {\prod }\limits_{x = 30}^{75} \left( {1 - {}_5q_x} \right)$$

Finally, we decomposed trends in CVD incidence and mortality using a decomposition technique featured in previous GBD publications^24,57^. In brief, we used a series of counterfactual analyses to break down the projected percentage change in CVD counts over 2020–2050 into three drivers: population growth, population aging and changes in age-specific rates. Because population growth and aging are major drivers of CVD trends but are largely non-modifiable, the objective of this analysis was to understand the extent to which blood pressure control could accelerate reductions in age-specific rates, counteracting the demographic drivers and reducing the growth rate in the number of individuals affected by CVD.

Specifically, we first calculated the number of new cases expected using 2020 population age structure and incidence rates applied to the total projected 2050 population. The difference between this number of expected cases and the number of projected cases from our model for 2020 gives us the change due to population growth. We then calculated the number of cases expected using 2020 death rates but using 2050 population age structure and 2050 total population projections. The difference between this number of expected cases and the number of projected cases from our model for 2020 gives us the change due to population growth and aging. Then, subtracting the change due to population growth from the change due to population growth and aging gives the change due to population aging alone. Lastly, the percentage change due to age-specific incidence rates is equal to the model-projected change in cases minus the percentage change due to population growth and the change due to population aging.

### Intervention implementation assumptions

Projected country-specific rates of scale-up of pharmacological treatment of raised blood pressure were informed by estimates of hypertension control over 1990–2019 as reported by the NCD Risk Factor Collaboration^18^. The metric of interest for our modeling was the proportion of individuals with hypertension whose blood pressure was at target—that is, ‘proportion controlled,’ sometimes referred to as ‘effective coverage’ of hypertension treatment. This metric corresponds to the product of the three levels of the hypertension care cascade: an 80-80-80 target would imply a control level (effective coverage level) of 0.80 × 0.80 × 0.80 = 51%.

We looked at trends in this metric across countries and over time to establish realistic bounds for the business-as-usual rate of scale-up of hypertension treatment. Supplementary Fig. 3 provides a plot of the incremental annual change in the proportion of individuals with hypertension whose blood pressure is controlled at different control levels for all countries included in the NCD Risk Factor Collaboration estimates. For countries with reliable data, trends in this metric generally fit a second-order polynomial pattern, with relatively faster increases in population blood pressure control at medium levels of control.

We defined our three scenarios in relation to country-specific estimates of blood pressure in 2019 and projected scale-up rates that followed the pattern described above. The business-as-usual projection was done as follows. A family of nine second-order polynomials were fit to different quantiles of ‘peak’ blood pressure control (Supplementary Fig. 4). Then, NCD-RisC data from 2010–2019 for each country were fit to each of the nine curves, and the model with the lowest squared error was selected to predict the scale-up pattern for 2020 onwards.

For the progress scenario, we assumed that each country would follow a scale-up pattern that slightly exceeded what had been achieved in the best-performing countries (for example, Canada, South Korea and Iceland). This function would imply a maximum scale-up rate of 3% per year and a ceiling effect (that is, no additional improvements in population control) at around 53% control, similar to what has been observed in the few countries already near or at the target. It follows that, given enough time, any country could achieve the 80-80-80 target. Whether the target could be reached by 2040 or 2050 would be determined by where along this curve a country lies, and countries with lower control levels in 2019 would take longer to get to 53% control.

For the ambitious scenario, we relaxed the assumption that blood pressure control would have a ceiling effect at 53% as described above. A recent review of historical hypertension programs^20^ and an interim analysis from WHO-HEARTS/Resolve To Save Lives initiative projects ongoing in 30 LICs and MICs^19^ suggest that 65–70% control is very possible. In addition, it has been noted that scale-up of anti-retroviral therapy for HIV/AIDS has generally progressed at about 5% per year globally^58^, representing a logistical and financial upper bound on what is achievable with current technologies and delivery systems. The aspirational scenario scale-up function was, thus, defined with an *x*-intercept (ceiling effect) reflecting 75% control and a peak scale-up rate of about 4% per year, slower than anti-retroviral drug therapy rollout but faster than the progress scenario.

The ambitious scenario is further corroborated by recent experiences with national HEARTS hypertension control programs. We analyzed internal data from the first 1–2 years of ten HEARTS programs that are already operating at scale in selected LICs and MICs. Rates of scale-up of effective coverage for hypertension in these country projects are included in Supplementary Fig. 3. The ambitious scenario assumes that the successes of the HEARTS program—or an alternative program with similar efficacy—could be replicated at scale in all countries.

Supplementary Fig. 5 plots the country-specific scale-up patterns that underlie the health effect modeling described previously. (We assumed no advancements in coverage between 2019 and 2022 largely due to the additional challenges and/or competing priorities that COVID-19 may be imposing on health systems during that period.) The year in which each country would achieve the proposed 80-80-80 target (main manuscript, Fig. 4) in each scenario is derived from Supplementary Fig. 5. The greater health effect of hypertension programs in LICs and lower-MICs in our analysis is largely due to their lower baseline control levels and greater scope for improving control over the time horizon of our analysis.

Implementation of sodium reduction policies was informed by WHO targets and recent country experiences. The business-as-usual scenario assumed that sodium intake levels would remain at their 2019 levels through 2050. The progress scenario was defined as a 15% reduction in sodium intake by 2030 relative to levels in 2023, the starting year for intervention implementation in our model. This degree of reduction was demonstrated within this timeframe in the United Kingdom and South Africa^59,60^, with South Africa having achieved the reduction over 3 years. The aspirational scenario was defined as a 30% reduction in sodium intake by 2027 relative to levels in 2023. This degree of reduction was demonstrated within this timeframe in South Korea^61^, which had a highly coordinated public health program that, among other features, targeted foods very high in salt, such as kimchi. We considered the South Korean experience aspirational because other countries might not have the same public health capabilities or ‘easy’ targets for reformulation. (In the absence of more up-to-date estimates, sodium intake levels in 2023 were assumed to be the same as in 2019.)

### Reporting summary

Further information on research design is available in the Nature Research Reporting Summary linked to this article.

## Online content

Any methods, additional references, Nature Research reporting summaries, source data, extended data, supplementary information, acknowledgements, peer review information; details of author contributions and competing interests; and statements of data and code availability are available at 10.1038/s41591-022-01890-4.

### Supplementary information

Supplementary Information Supplementary Figs. 1–5

Reporting Summary

## Figures and Tables

**Extended Data Table 1:**
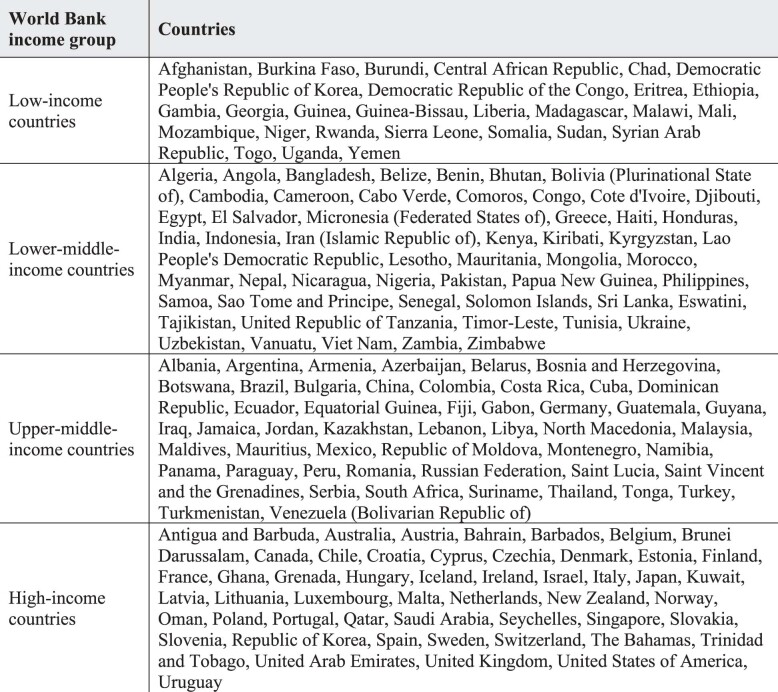
Countries included in this analysis. Classifications for each country are based on the World Development Indicators 2021 thresholds based on gross national income per capita estimates for each country

## Data Availability

All data used as inputs to our modeling analyses are publicly available from their respective organizations (for example, NCD Risk Factor Collaboration). Processed versions of these data are contained in model input files located at https://github.com/Disease-Control-Priorities/HTN. All output data (results) from this study are available for download through the visualization tool located at https://dcp-uw.shinyapps.io/bp-targets.

## Code availability

All analyses were performed using R version 3.6.1. Scripts are available through https://github.com/Disease-Control-Priorities/HTN.
