# Supplementary material for: Modeling global 80-80-80 blood pressure targets and cardiovascular outcomes
Source: Nat Med. 2022 Jul 18;28(8):1693–9. doi: 10.1038/s41591-022-01890-4 (PMC9388375; doi:10.1038/s41591-022-01890-4)

nature portfolio

https://doi.org/10.1038/s41591-022-01890-4

Supplementary information

Modeling global 80-80-80 blood pressure targets and cardiovascular outcomes

In the format provided by the authors and unedited

Supplementary Figure 1. State-transition model schematic example for ischemic heart disease (IHD).

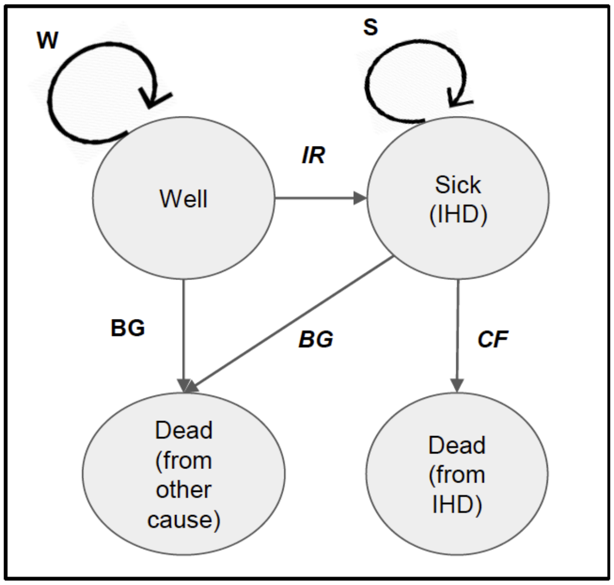

Variable	Description
**W**	Probability of remaining in the well state
**S**	Probability of remaining in the sick state
**BG**	Probability of dying from another cause (not IHD in the figure above)
**IR**	Probability of becoming sick
**CF**	Probability of dying from the model-specific cause

Supplementary Figure 2. Intervention effect model. Population blood pressure distributions for each age and sex combination in each country were mapped to the six bins shown in the figure, which were then compared to observed CVD incidence rates to estimate blood pressure level-specific CVD incidence using relative risk estimates from the literature and Equations 7-9 in the Online Methods. The intervention effect modeling was operationalized by shifting population blood pressure distributions downwards (i.e., higher proportions in lower blood pressure bins) and recalculating CVD incidence rates using Equations 7-9, holding age-sex-country-specific y_i and α parameters constant.

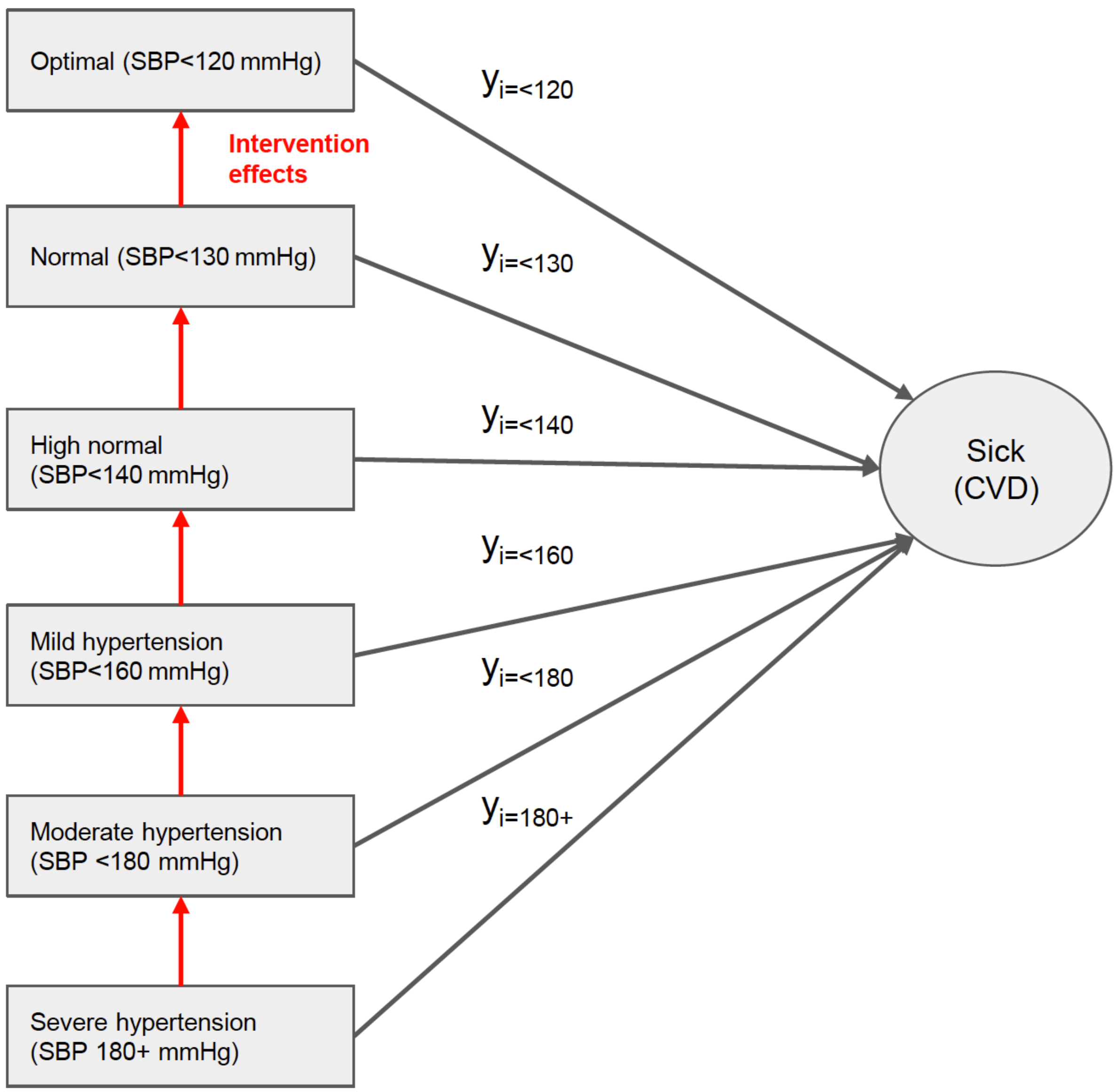

Supplementary Figure 3. Scale-up functions assumed in the progress and aspirational scenarios. As described in the Online Methods, we used historical data for 182 countries over 1990-2019 from the NCD Risk Factor Collaboration (black points on the plot below) to establish a plausible upper limit (frontier) to the annual rate of increase in population blood pressure control (y axis) at any given observed level of population blood pressure control (x axis). For simplicity of modeling, the frontier was parameterized using a quadratic function (red curve). Of note, trends in high-performing countries suggest no further improvements in control beyond a control level of about 50-55%, so in the progress scenario a ceiling effect was assumed. Our aspirational scenario relaxes this assumed ceiling effect to 75%; i.e., no more than three in four individuals with hypertension at optimal blood pressure (blue curve).

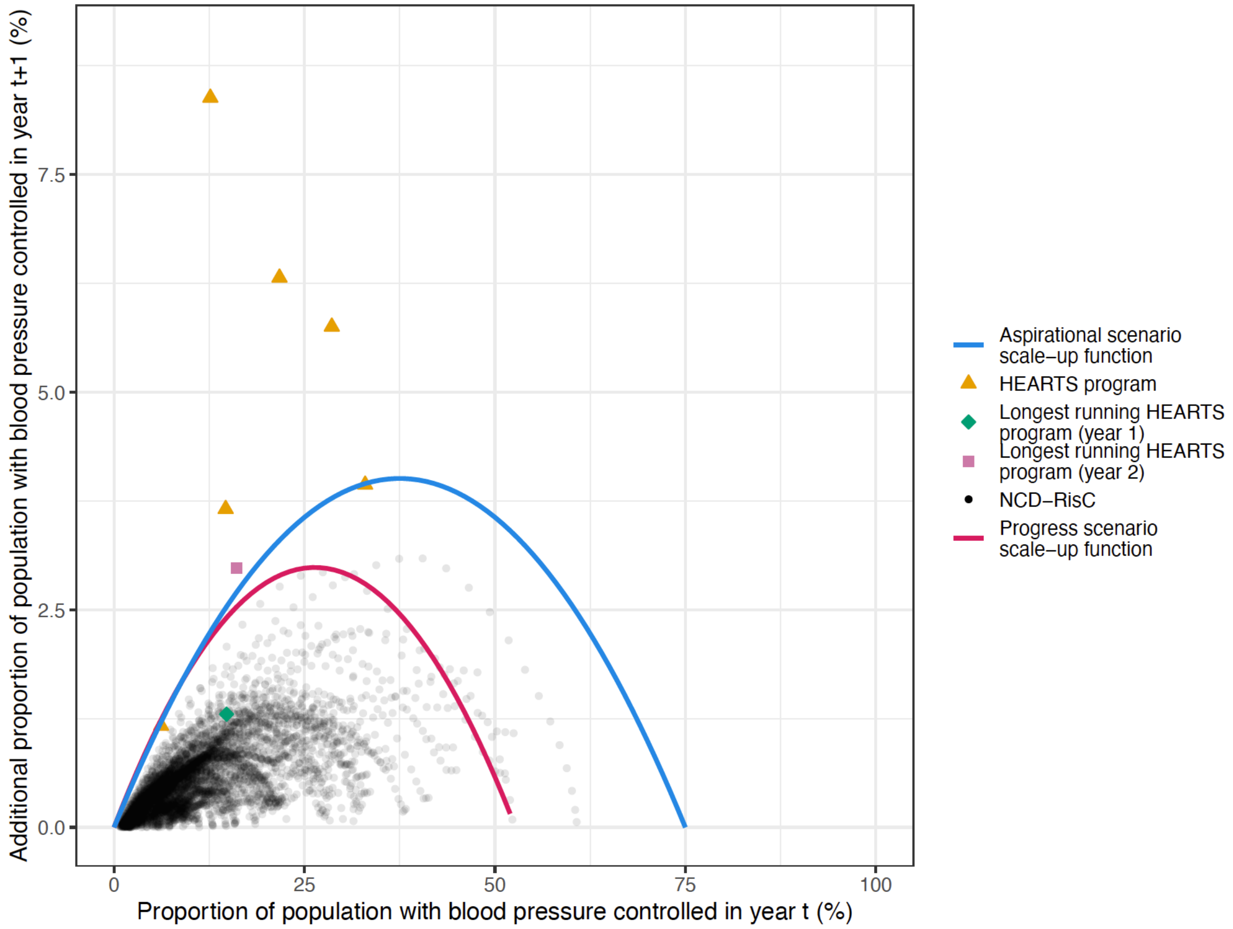

Supplementary Figure 4. Approach to projecting improvements in blood pressure control in the business-as-usual scenario. The panels below show the polynomial functions used to project hypertension control (yellow lines), with country data added to illustrate fit. For simplicity, only three of the nine functions are shown, representing the best-, middle-, and worst-performing countries.

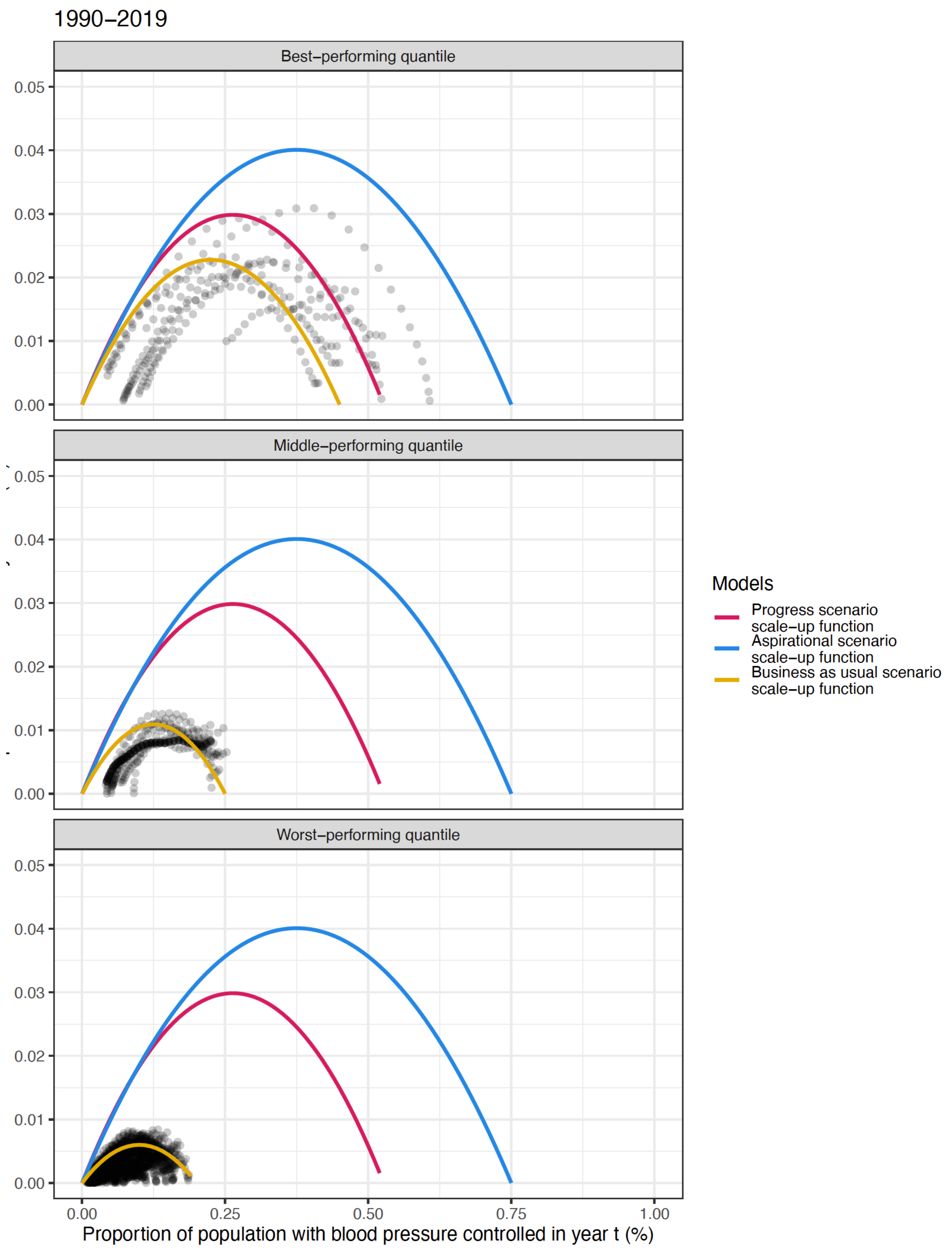

Supplementary Figure 5. Scale-up patterns for hypertension treatment, by intervention scenario (columns) and country income group (rows). As described in the Online Methods and Extended Data Figures 5-6, we used a second-order polynomial-based scale-up function to model future changes in population blood pressure control levels; this produces a cubic pattern when the treatment intervention is scaled-up over time. The first portion of each plot shows historical trends for each country as per the NCD Risk Factor Collaboration estimates. Beyond 2022, control levels evolve according to the scale-up function specified in each of the three scenarios. The red dotted line in each plot denotes the proposed 80-80-80 target (i.e., 51% hypertension control).

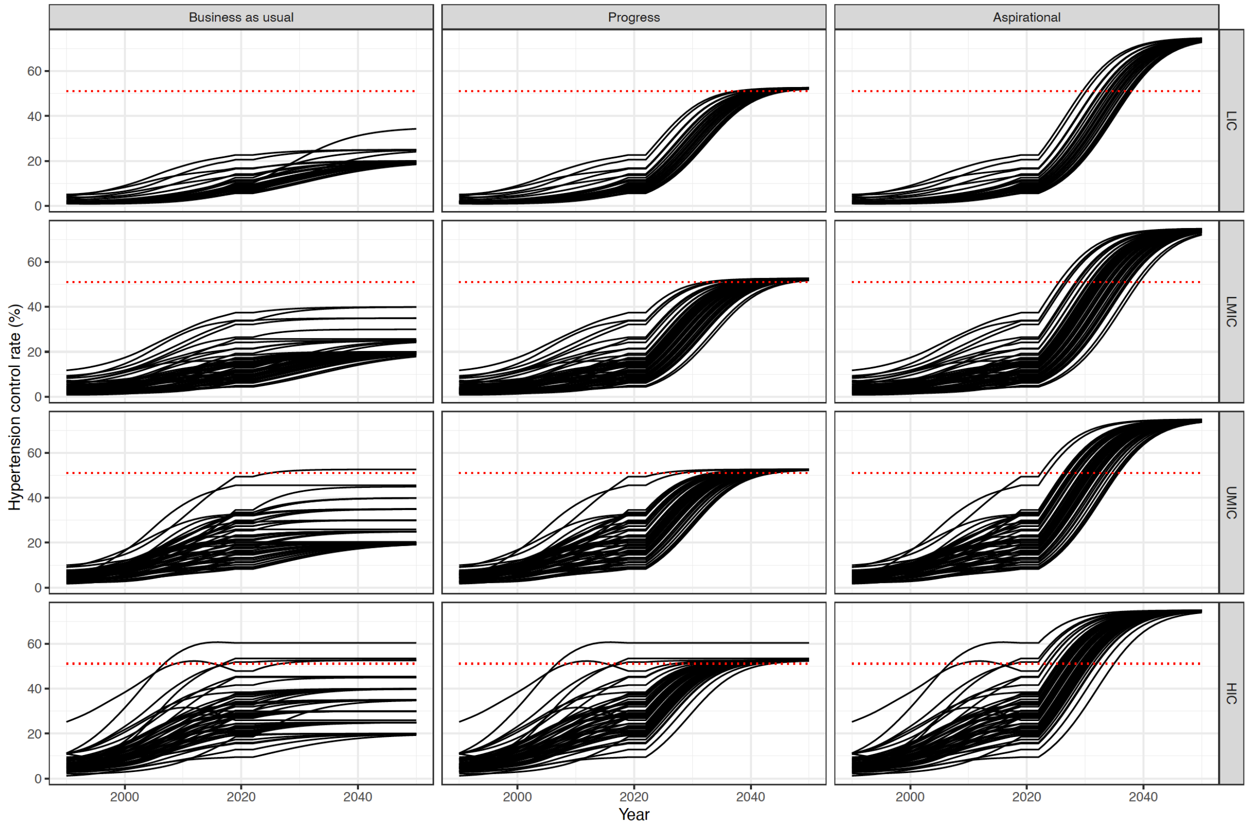

Supplement: Supplementary file 1 — Supplementary Figs. 1–5 [file 41591_2022_1890_MOESM1_ESM.pdf]
